# Potential Complications of Percutaneous Vertebroplasty Combined with Interstitial Implantation of ^125^I Seeds for Metastatic Spinal Tumors: A Case Report and Literary Review

**DOI:** 10.1155/2019/1328172

**Published:** 2019-08-28

**Authors:** Lunli Xie, Zhenlin Yan, Jun Zhu, Xudong Chen, Changyuan Yang, Xing Su, Keqin Quan, Dan Pu

**Affiliations:** ^1^The Minimally Invasive Department of Orthopedics, Rehabilitation Medical Center, The First People's Hospital of Huaihua, Hunan, Huaihua 418000, China; ^2^The Department of Sport & Rehabilitation Medicine, Institution of Orthopedics, Medical School of Jishou University, Hunan, Jishou 418000, China

## Abstract

Percutaneous vertebroplasty is often used to acquire the stability of the spine and relieve the pain caused by osteoporotic vertebral compressive fracture (OVCF). ^125^I seeds have been used for application of local therapy for tumors. A combined treatment was reported in previous literatures. Thus, this case report is aimed at reporting a patient with an occurrence of delayed radioactive myelopathy, who accepted percutaneous vertebroplasty combined with interstitial implantation of ^125^I seeds for metastatic spinal tumors, and at reviewing the published literatures.

## 1. Introduction

The majority of metastatic spinal tumors occur in the thoracic spine, followed by the lumbar spine and the cervical spine [1]. The vertebral body is easily damaged by spinal metastatic tumors due to its large volume and the abundance of blood vessels within it. Besides, spinal metastasis tumor usually spreads in the vertebral body with aggressive behavior, leading to adverse events such as severe pain, spinal deformity, spinal instability, and even neurologic dysfunction [2]. Thus, various therapeutic methods such as conservative treatments and surgery have been used to treat metastatic spinal lesions [3]. However, the conservative methods including radiotherapy and chemotherapy are limited due to contraindication such as vertebral fractures and spinal instability. Indeed, the patients with metastatic tumors accepted traditional open surgery which always requires long-term postoperative recovery period, often increases mortality, and usually causes delayed treatment for primary tumors [4]. Therefore, the minimally invasive surgical method with percutaneous vertebroplasty (PVP) has been accepted by the surgeon and patient because of nonvascular intervention in injury and reliving of back pain from vertebral body fractures.

Recently, a novel composite therapeutic method defined as the PVP plus interstitial implantation of ^125^I seeds has been used to copy vertebral body fractures caused by metastatic tumors, and many published literatures have also proved the effectiveness and safety of this combined way [5]. Nevertheless, the potential radioactive myelopathy, in fact, proved by basic research, caused by ^125^I seeds should be paid more attention by the surgeon in spite of the lack of powerful evidence originating from clinical study. Herein, in the present article, we report a case with radioactive myelopathy and review the published literatures.

## 2. Case Report

The informed consent was provided by the patient, and ethical approval was warranted by the First People's Hospital of Huaihua.

**Figure 1:**
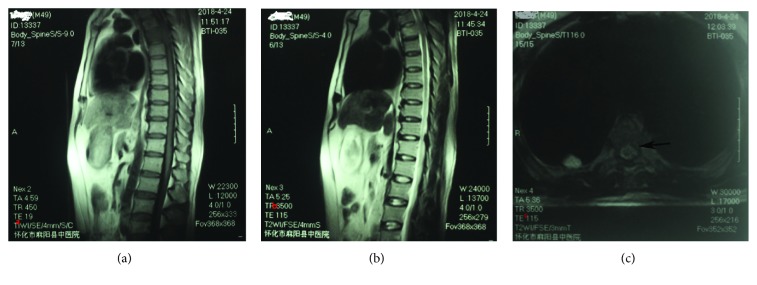
The preoperative thoracic magnetic resonance imaging (MRI) showed a minor intraspinal occupying-lesion and associated pathological fractures of the vertebrae. (a, b) The T1-weighted and T2-weighted images show the fractures of the thoracic vertebrae body. (c) The cross section shows a possibility of an intraspinal space-occupying lesion in the T4 level like the black arrow indicated.

**Figure 2:**
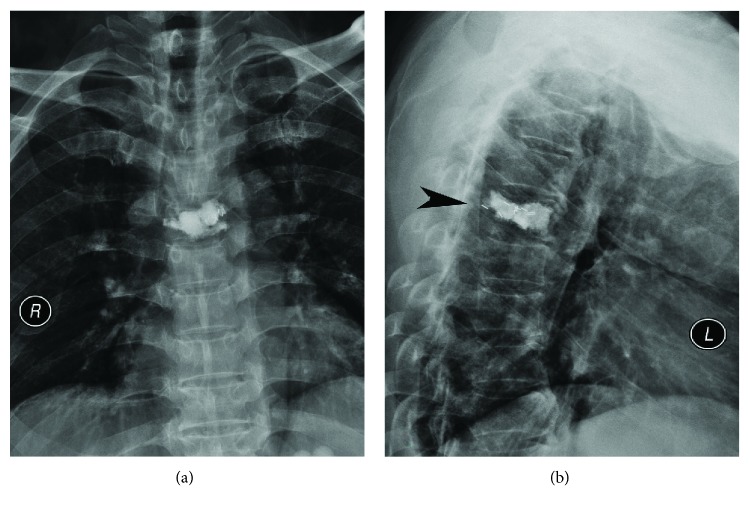
(a, b) The regular films of X-ray about the thoracic vertebrae show bone cement plus ^125^I seeds in the T4 body. Black arrow indicates the location of ^125^I seeds close to the back wall of the vertebra.

**Figure 3:**
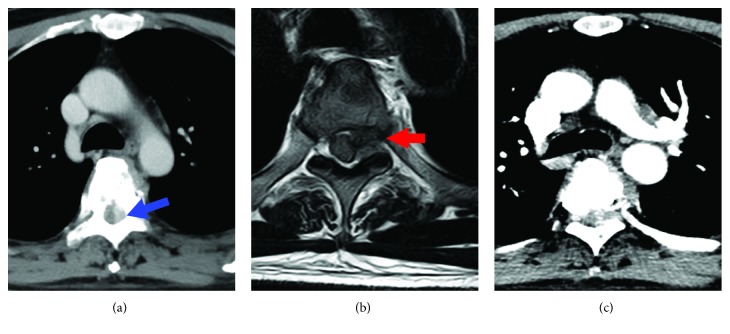
(a) The postoperative CT shows bone cements and metallic implants in T4 without any leakage in the T4 vertebrae body. (b) The postoperative MRI shows a larger intraspinal space-occupying lesion in the T4 intraspinal level. (c) The enhanced CT indicated the possibility of tissue tumor. The blue arrow and red arrow indicate the possibility of tissue tumor or hematoma.

**Figure 4:**
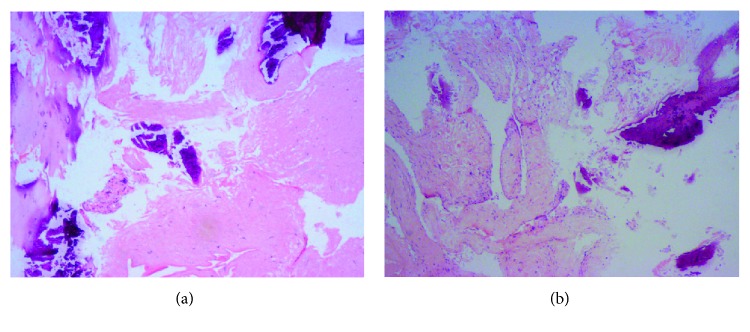
(a, b) Photograph of the tissues of intraspinal space-occupying lesion and are H&E ×100 pictures. These show that the fiber tissues keep their normal shape without any discovery of tumor cells.

**Figure 5:**
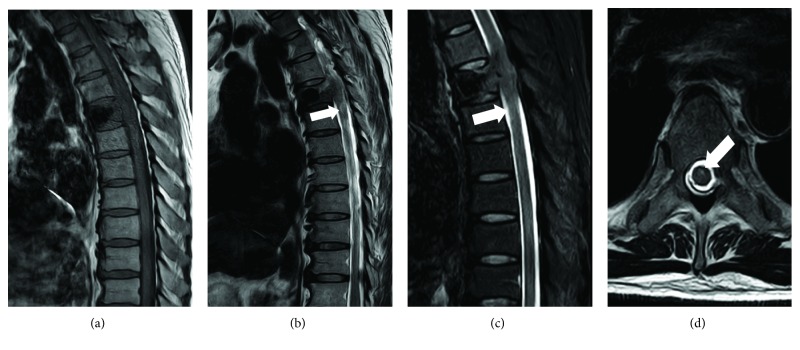
(a) T1-weighted image shows that the T4 body was filled with PMMA and ^125^I seeds. (b–d) The high signal intensity of the spinal cord in T2-weighted and fat-suppression images in the T5 level. The white arrow shows the high signal intensity of the spinal cord.

A 49-year-old man presented with a 5-month history of back pain who was diagnosed with fourth thoracic vertebral body fracture caused by tumor cells based on pathology results. The tumor cells in the fourth thoracic vertebral body originated from hepatocellular carcinoma. The preoperative thoracic magnetic resonance imaging (MRI) showed a minor intraspinal space-occupying lesion in the fourth thoracic vertebra canal and found pathological fractures of the fourth vertebrae (Figure 1). Back pain as the only clinical symptom without any weakness, numbness, or other symptoms of spinal cord injury in both lower extremities was reported to the physician. And then, he accepted the percutaneous vertebroplasty (PVP) plus interstitial implantation of ^125^I particles (10 particles) at a local county hospital (Figure 2). Back pain was relieved postoperatively, 3 days after he received PVP combined with interstitial implantation of ^125^I seeds. However, back pain, bilateral lower extremity weakness, and loss of bladder control reoccurred on day 3 after surgery and got worse. A general physical examination and central nervous system examination upon patient admission revealed abnormal findings. The majority of symptoms encompassed pain and hypoesthesia below the processus xiphoideus. The abdominal reflex, the crissum and cremasteric reflex, and the knee and ankle reflex could not be induced. No pathological reflection of Babinski's sign was induced. The strengths of the major muscle of both lower limbs were 3 grades and progressively decreased in the postoperative period. The opiate drugs were used to control pain originating from the surgical site. He was admitted to our hospital after 44 postoperative days because of serious back pain. The postoperative transverse computed tomography (CT) revealed bone cements and metallic implants in T4 without any bone cement leakage, but the MRI showed the left intraspinal space-occupying lesion in the T4 vertebral body (Figure 3). And the enhanced CT indicated the possibility of tissue tumor, which was also confirmed by thoracic vertebra magnetic resonance imaging (Figure 3). However, no powerful evidences could be used to confirm that the intraspinal space-occupying lesion was a tissue tumor (Figure 4). In fact, the results of MRI showed the high signal intensity in T2-weighted and fat-suppression images in the T5 body level (Figure 5). Notably, there are several particles located in the vertebral posterior wall without appropriate distribution (Figure 2). The above clinical symptoms may be caused by delayed radiation-induced myelopathy or spinal cord compression. This patient died after 5 months of surgery because of multiple organ failure.

## 3. Discussion

Spinal metastasis tumor usually causes pathological fracture of the vertebral body leading to the compression of the spinal cord and resulting in severe pain or limb sensory and motor disorders. These can seriously decrease the quality of life of tumor patients even if these populations have short-term life expectancy [6]. The basic therapeutic rationales include relieving pain, rebuilding the stability of the spine, improving the quality of life, and killing the local tumor cell as soon as possible. However, traditional surgical methods and chemotherapy have many defects, such as major trauma, long bedtime rest, long cycles, and significant complication and severe adverse effects [7]. The percutaneous vertebroplasty (PVP) as palliative care can relieve the pain and rebuild the stability for pathological fracture of the vertebral body [8]. Besides, PVP can also kill the tumor cells in virtue of high temperature produced by polymethylmethacrylate (PMMA). However, this method can kill only tumor cells surrounding the PMMA and cannot provide ongoing kill ability [4]. Thus, some researchers combined PVP with the interstitial implantation of ^125^I seeds in order to acquire ongoing kill ability to tumor cells [4, 5, 9–11]. For one thing, this novel technique provides stronger anchoring and fixation with the help of PMMA. For another, tumor cells in the diseased vertebral body can be continually killed by gamma rays produced by ^125^I seeds. The feasibility of this comprehensive method had been proved by a previous physician. But there are limitations of this novel measures because of many potential complications especially delayed radiation-induced myelopathy and intraoperative leakage of bone cement [12, 13].

The relative published literatures indicated that no radioactive myelopathy occurred in the patients who accepted PVP plus interstitial implantation of ^125^I seeds [5, 6, 9–11]. We searched PubMed with the following terms: “percutaneous vertebroplasty or PVP” AND “^125^I seeds” with limitation scope of spinal metastasis tumor and clinical research, and then, we found 5 literatures including 4 clinical studies [5, 6, 10, 11] and 1 case report [9]. The characteristics of the published articles were summarized in Table 1. There are few clinical articles that reported radioactive myelopathy but for bone cement leakage for a patient accepting this novel surgery to therapy spinal metastasis.

**Figure 6:**
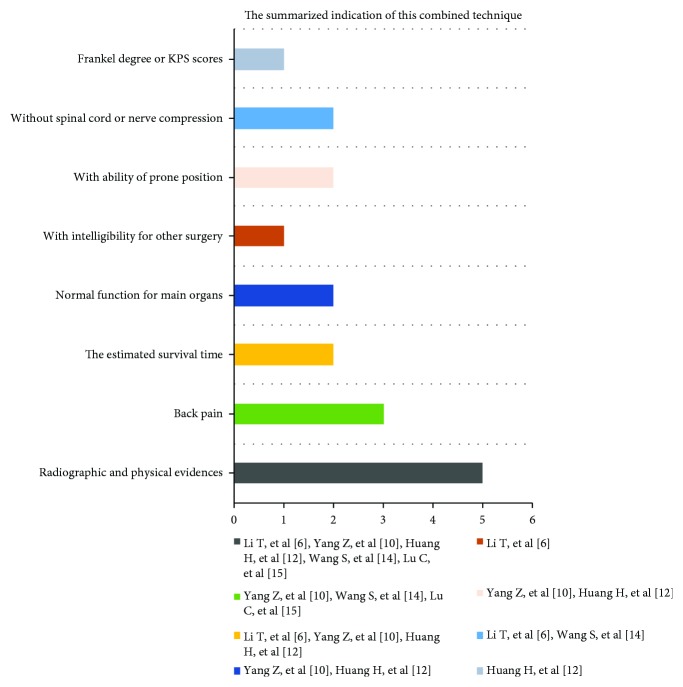
The summarized indications of the PVP plus interstitial implantation of ^125^I particles. The majority of indications of the PVP combined with interstitial implantation of ^125^I particles include the definite radiographic and physical evidence, back pain as the major clinical symptom, and with more than three months of the estimated survival time. The second indications consist of normal function of main organs, with ability of prone position (more than 2 hours), and without spinal cord or nerve compression.

Many factors such as the type of fracture, surgical technique, surgeon's experiences, suitable implanting location, and proper dose of ^125^I particles are usually the key to increase the therapeutic efficacy of this combined surgery and reduce risks, especially the suitable implantation location and fitted dose of ^125^I particles that may bring better therapeutic effectiveness and can avoid radioactive myelopathy, which have been emphasized in previous published literatures [6, 10, 12, 14, 15]. Thus, the surgeon should have skilled technique and rich experience of PVP. Besides, the performer must elaborate the indications of this combined technique and should strictly follow the treatment planning system (TPS). TPS and CT three-dimensional (3D) digital image reconstruction are used to perform evaluation and analysis before this novel surgery. In a previous case report, the surgeon used a TPS software and a 3D digital image to evaluate absorbed dose values based on lesion size and location and analyzed the relationship with surrounding normal tissues [9]. In fact, the dose of ^125^I particle implantation can be calculated by the surgeon according to 3D icons, isodose curve, absorbed dose value, and relationship with surrounding normal tissues. Our patient accepted the percutaneous vertebroplasty (PVP) plus interstitial implantation of ^125^I seeds (10 particles) at a local county hospital who has an occurrence of delayed radioactive myelopathy. The postoperative X-ray showed that the location of a part of ^125^I particles was close to the back wall of the vertebra (Figure 2), which may be the cause of delayed radioactive myelopathy. Thus, the suitable location of implantation with the help of preoperative evaluation and the effective anchoring by means of bone cement are necessary for the surgeon in operative process. Besides, surgeons should strictly follow the indications of this novel surgery. After reviewing five related published clinical studies [6, 10, 12, 14, 15], the following seven main items of indications for PVP plus implantation of ^125^I particles had been summarized (Figure 6). Firstly, the definite radiographic and physical evidences to confirm spinal metastasis were emphasized in five clinical studies [6, 10, 12, 14, 15]. Secondly, three literatures revealed that back pain with or without neck pain should be considered as the major clinical symptom [10, 14, 15]. Thirdly, other indications are as follows: with more than 3 months of the estimated survival time [6, 10, 12], with normal function of main organs [10, 12], with ability of prone position (more than 2 hours) [10, 12], and without spinal cord or nerve compression [6, 14], which were presented in four published clinical studies. Finally, patients with intelligibility for other surgery and their Frankel degree or KPS scores in preoperative duration were also viewed as indications for this combined surgery [6, 12]. According to the above indications of this novel surgery, the main indications include the definite radiographic and physical evidence, back pain as the major clinical symptoms, and patient with more than three months of the estimated survival time. The second indications consist of normal function of main organs, with ability of prone position (more than 2 hours), and without spinal cord or nerve compression. The patient was suitable for receiving PVP combined with implantation of ^125^I particles based on the above indications in our report.

Notably, it is difficult to confirm the radiation-induced myelopathy because of unspecific features in clinical and radiological findings [16, 17]. Our report describes a patient with the possibility of postoperative radioactive myelopathy who accepted PVP plus interstitial implantation of ^125^I seeds as before. The evidences were derived from the bilateral progressiveness of clinical symptoms, negative pathological examination of an intraspinal space-occupying lesion, and the high signal intensity in T2-weighted and fat-suppression images. Actually, the unilateral compression of the spinal cord always leads to the appearance of the Brown-Sequard syndrome rather than bilateral clinical symptoms for both lower extremities. In particular, the improper distribution of ^125^I particles was found in radiography, which might cause the delayed radioactive myelopathy (Figure 4). In basic researches, actually, the autophagy of neural cells was found in Banna pigs which received ^125^I seed implantation that was demonstrated by previous researchers [18, 19]. And ultimate apoptosis and necrosis were also observed in the pathological section. In an in vivo study, electron microscopic (EM) observation was performed for subcellular identification. Under EM observation, the obvious impairment was found for most organelles, mitochondria swelled, and apoptosis of neurons was found as well [20]. Based on that, the authors suggested that radiation myelitis due to ^125^I-based brachytherapy is related to the dose and duration of exposure. Besides, all documents in published articles emphasize the safe distance (greater than 1 cm) ranging from the particles to the spinal cord or vessels [5, 6, 9–11].

## 4. Conclusion

The case report describes a 49-year-old man who presented with a possibility of delayed radiation-induced myelopathy after he had accepted PVP combined with local ^125^I seed implantation. An adverse circumstance occurred which was compared with previous conclusion results from published papers. The complication of radioactive myelopathy or delayed radioactive myelopathy should be paid enough attention by the surgeon. In particular, when the surgeon aims to acquire effective particle distribution, the surgeon should not overlook the safe distance ranging from particles to the spinal cord.

## Figures and Tables

**Table 1:** The published literatures of novel therapeutic methods for spinal metastatic tumors.

Year and author	Size of sample		Age (years)	Follow-up time (months)	Dosage (seeds per patient)	Tools of evaluation	Complications	Therapeutic effects	
	Male	Female							
								Clinical states	Radiological manifestations
2009, Yang et al. [5]	21	19	60.95 ± 4.48	12	2.92^∗^ 5–10	VAS, KPS, RECIST, CT, MRI	Bone cement leakage	Alleviate back pain Improve quality of life	Acquire spinal stability Prohibit and kill tumor cells
2011, Zuozhang et al. [9]	1	—	38	36	2.92^∗^	VAS, KPS, RECIST, CT, MRI	No complications		
2012, Yang et al. [10]	20	30	61.14 ± 5.12	12	7–20 80–100^#^	VAS, QLQ-C30, CT, MRI	Low blood pressure Low oxygen saturation High level of thromboses		
2014, Li et al. [6]	18	11	49.2	3	90–140^#^	VAS, KPS, RECIST, CT, MRI	Bone cement leakage		
2014, Huang et al. [11]	10	8	62	6	16-34	VAS, KPS, ECT, CT	Bone cement leakage		

^∗^cGy/hour/seed; ^#^Gy.
